# Establishment of an image evaluation grading criteria for experimental stifle joint osteoarthritis in dogs: an X-ray and CT imaging study

**DOI:** 10.1186/s42826-023-00186-z

**Published:** 2023-12-15

**Authors:** Beomseok Rhee, Changfan Jin, Seo-Hyun Shin, Hojung Choi, Youngwon Lee, Sokho Kim

**Affiliations:** 1 Research Center, HLB BioStep Co., Ltd., Incheon, 22014 Republic of Korea; 2 Department of Veterinary Medical Imaging, College of Veterinary Medicine, Chungnam National University, Daejeon, Republic of Korea

**Keywords:** Osteoarthritis model, Image evaluation grading criteria, Dog, Stifle joint

## Abstract

**Background:**

This study aimed to establish an image evaluation grading criteria for experimental stifle joint osteoarthritis (OA) in anterior cruciate ligament transection induced OA beagle dog models. The severity of OA was assessed using X-ray and computed tomography (CT) imaging.

**Results:**

A total of 32 dogs (8 controls and 24 OA-induced dogs) were included in the study. The OA-induced group showed significantly higher manual joint palpation, gait analysis, and OA severity scores than the control group. Based on these two results, we calculated correlation coefficients. There was a strong positive correlation between manual joint palpation scores and OA severity on diagnostic imaging and between gait analysis scores and OA severity.

**Conclusions:**

The developed grading criteria based on radiographic evaluation correlated with clinical assessments. The study also employed CT imaging to enhance the accuracy and sensitivity of early-stage OA change detection in the stifle joint. However, further studies with larger sample sizes and multiple evaluators are recommended for the validation and generalizability of this grading system. These established image evaluation grading criteria can help evaluate and monitor the efficacy of interventions and changes in OA lesions in canine models.

## Background

Anterior cruciate ligament transection (ACLT) stifle joint osteoarthritis models are a useful way to study the pathogenesis of arthritis and evaluate the effectiveness of drug treatments [1]. The methods for assessing changes in these arthritis models include X-ray imaging, examination of synovium, biochemistry, histochemistry, and macroscopic evaluation. Radiological images are useful for identifying pathological changes in patients with arthritis and are increasingly used because they are noninvasive and allow for serial evaluation. Although many methods are used for radiological assessment of arthritis, it is still necessary to grade images of knee arthritis to quantify the severity and compare changes.

To quantify the changes seen on radiographs, grading systems have been developed for human knee osteoarthritis and to a lesser degree for canine stifle osteoarthritis [2]. In humans, the Kellgren–Lawrence (KL) grade via X-ray has been used for a long time for several joints [3], but unlike humans, dogs cannot be X-rayed under weight-bearing conditions; thus, there is need to change the KL grade evaluation methods for application in dogs.

Grading of the hip joint in dogs has been previously studied [4] and is possibly applicable to knee joint evaluation after development. The knee joint in dogs is too small to be evaluated by X-ray alone as in humans. Therefore, it is possible to obtain cross-sectional images, which eliminates the difficulty in reading due to overlapping by adding CT imaging to the evaluation tools. This study aims to demonstrate the necessity of CT imaging evaluation as a complement to X-ray evaluation for assessing OA in experimental stifle joint osteoarthritis model dogs and will provide corresponding grading criteria.

## Results

As shown in Table 1, both groups, the control group and the experimental group, were evaluated using manual palpation, gait analysis, and OA severity with X-ray and CT imaging. Manual palpation analysis using a score ranging from 0 to 4 points, where higher scores represent more severe pain. The manual joint palpation score in the OA group was significantly higher than that in the control group (*P* = 0.001). Additionally, limb function of the control group and OA group was evaluated using gait analysis using a score ranging from 0 to 4 points, where higher scores represent impaired gait. The gait analysis score in the OA induction group was significantly higher than that in the control group (*P* = 0.001).

**Table 1:** Cases of scores in manual joint palpation, gait analysis, and OA severity were examined

	Control group (n = 8)	Experimental group (n = 24)
*Manual joint palpation (score)*		
0	8	0
1	0	0
2	0	7
3	0	13
4	0	4
*Gait analysis*		
0	8	0
1	0	2
2	0	5
3	0	13
4	0	4
*OA severity*		
0	8	0
1	0	1
2	0	10
3	0	13
4	0	0

**Fig. 1:**
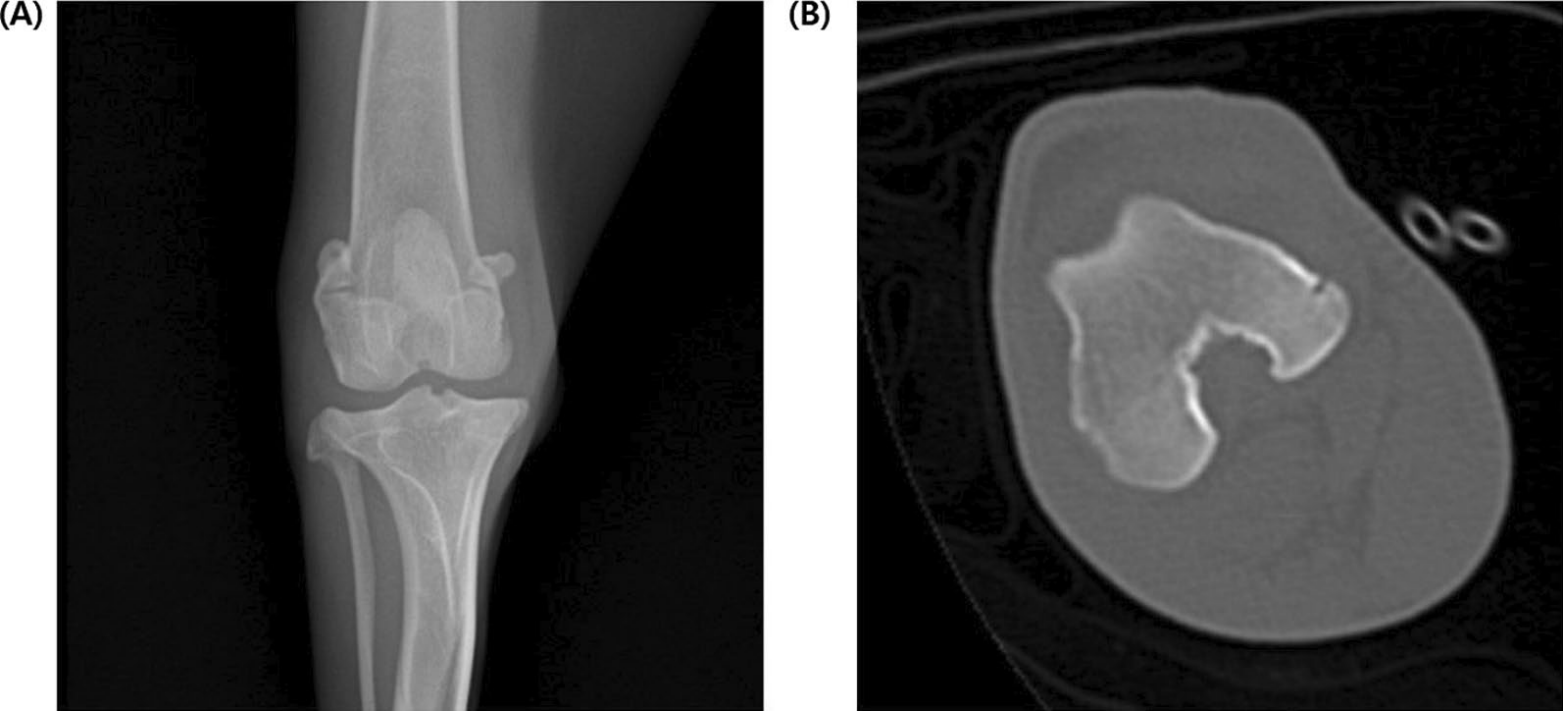
X-ray (**A**) and computed tomography (CT) (**B**) for early osteoarthritis of the stifle joint. Even in the case of the initial change in osteoarthritis, which is hard to detect in X-ray images (small osteophytes, etc.), it can be detected in CT images.

**Fig. 2:**
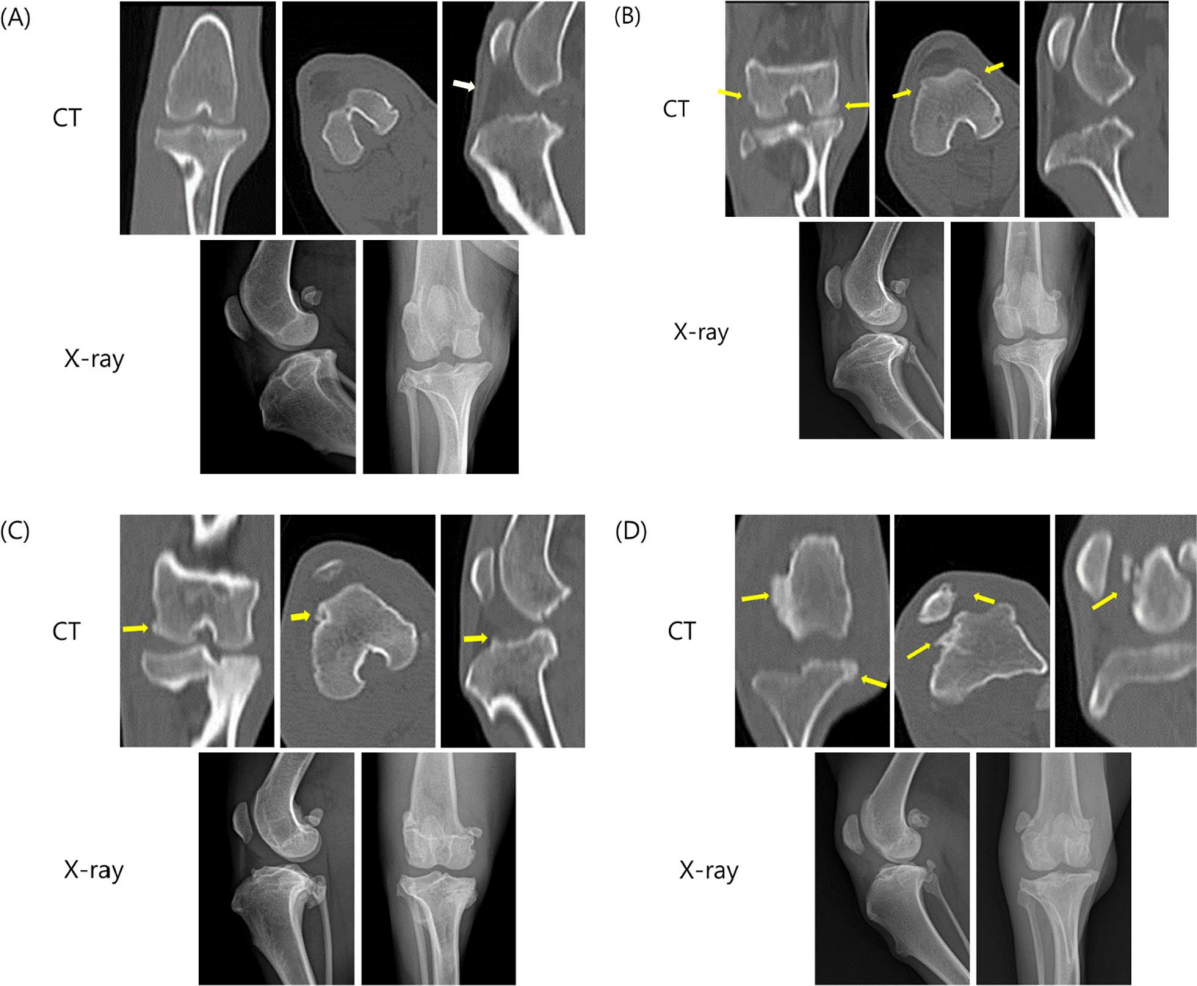
Representative images of OA severity using computed tomography(upper) and X-ray(lower) imaging. Validation results by criteria for assessing OA severity using both CT and X-ray. OA severity was assessed using both CT and X-ray scores ranging from 0 to 4 points, **A** Score:1; **B** Score:2; **C** Score:3; **D** Score:4, with higher scores representing greater severity. The severity score in the OA group was significantly higher than that in the control group (*P* = 0.001). The yellow arrow indicates an osteophyte. The white arrow indicates a doubtful opacity in the infrapatellar fat pad

As shown in Fig. 1, we conducted X-ray and CT imaging in the OA model group. All animals in the OA group had increased infrapatella opacity and osteophytes, which are analogous to the typical symptoms found in human OA patients with KL grades 2–3. Interestingly, changes indicative of early-stage OA, such as small osteophytes, were not visible on X-rays but were observed on CT images. Therefore, we validated criteria for assessing OA severity using both X-ray and CT. OA severity was assessed using both CT and X-ray scores ranging from 0 to 4 points, with higher scores representing greater severity (Fig. 2). The severity score in the OA group was significantly higher than that in the control group (*P* = 0.001).

To validate the integrity of image evaluation scoring in the OA model, we calculated the correlation coefficient between the previously conducted behavioral evaluation scoring and image evaluation scoring (Table 2). There was a strong positive correlation between manual joint palpation and OA severity (r = 0.695; *P* < 0.01). Additionally, there was a strong positive correlation between gait analysis and OA severity (r = 0.592; *P* < 0.01).

**Table 2:** Correlation between manual joint palpation, gait analysis and OA severity

	Manual joint palpation	Gait analysis	OA severity
Manual joint palpation	1		
Gait analysis	0.690**	1	
OA severity	0.695**	0.592**	1

**P < 0.01

## Discussion

Canine joints have mechanical properties and morphological structures similar to human joints. Additionally, canine joints are large enough to allow multiple physical and functional assessments. Beagles are a dog breed suitable for animal testing and are widely used in studies. Canine anterior cruciate ligament transection (ACLT) models are the most widely used models for joint instability. Surgically-induced canine OA models have been studied for decades and are considered to be the best available OA model due to their similarity with OA in humans [5]. This model relies on a combination of joint instability, altered joint mechanics (i.e., changed load bearing or joint congruence), and inflammation to induce OA lesions. The model then imitates degenerative joint disease that is characterized by progressive loss of articular cartilage and subchondral bone changes with joint space narrowing, subchondral bone sclerosis, and osteophyte formation, which closely mimics the progression of OA in humans. However, the progression of disease in surgically induced models (including ACLT models) is much more rapid than that in human OA, which is caused by aging due to mechanical stress [6–8]. Dogs often shift their weight to the unoperated leg due to pain in the OA-induced leg, which can disrupt progressive joint degeneration in the operated leg. To avoid inhibiting the progression of joint degeneration, ACLT is also performed on both joints [9, 10]. OA is a multifactorial disease associated with mechanical loading, fluctuations in hormonal levels, and modulation of nervous system pathways [11, 12]. Most OA related studies in animal models have been performed with males [13], to avoid variability caused by the estrous cycle in females.

In humans, OA is usually diagnosed and analyzed using X-ray imaging. Although the anatomy of the canine stifle joint is similar to that of the human knee joint, it is statistically smaller. The radiographic grading system to determine OA severity has been optimized for the human knee joint but has been less developed for the canine stifle joint [2]. Therefore, a system more sophisticated than X-ray would help diagnose canine stifle joint conditions with better accuracy and reliability. Computerized tomography, a form of advanced radiography, provides higher resolution images and allows both 2D and 3D reconstructions of bony features. Thus, CT imaging can detect subtle bony lesions typically found in the early stages of OA with greater sensitivity, which may not be possible with traditional radiography (Fig. 1) [14].

The measurement of joint space narrowing and osteophytes on radiographs of weight-bearing knees is a standard approach to assess OA progression in humans. Based on these measurements, Kellgren and Lawrence established a radiographic classification scheme for OA [15]. Currently, KL classification is the most widely used clinical tool for the radiographic diagnosis of OA [3]. KL grading is conducted on radiographs of the knee using the coronal view of weight-bearing semiflexed knees. Since the beagle dogs were under anesthesia when the radiographs were taken, the limbs were not weight-bearing, making it difficult to directly apply the KL grading system. Therefore, the severity of the disease was evaluated by determining osteophyte formation and the degree of opacity of the infrapatellar fat pad, instead of directly measuring joint space narrowing. Radiographic infrapatellar fat pad analysis is usually used in veterinary practice to diagnose degenerative joint conditions in dogs (Fig. 2) [16].

In this study, we created a canine OA model through a surgical procedure and evaluated it four weeks later using manual joint palpation evaluation, gait analysis, and radiographic imaging (Table 1). As shown in Fig. 1, changes indicative of early-stage OA, such as small osteophytes, were not visible on X-rays but were observed on CT images. Therefore, we validated criteria for assessing OA severity using both X-ray and CT (Table 5). Moreover, we calculated the correlation coefficient between the previously conducted behavioral evaluation scoring and image evaluation scoring (Table 2). Correlation analysis is a method used in probability and statistics to analyze the linear relationship between two variables [17]. Both manual joint palpation and gait analysis confirmed a strong positive correlation with OA severity. This implies that the radiographic evaluation criteria are highly related to the behavioral evaluation criteria, manual joint palpation, and gait analysis when they align with each other.

Healthy normal stifle joints are classified as grade 0 during manual joint palpation evaluation, gait analysis, and OA severity. However, ACLT-induced stifle joint osteoarthritis has increased grading scores. Radiography was not only useful for identifying changes of osteoarthritis, but also provided a noninvasive, serial method of evaluation [1]. The imaging grading system made in the present study was correlated with manual joint palpation and gait analysis, which may be used to classify lesion changes using the grading score. Nevertheless, follow-up studies with larger samples are needed, as the present study was not cross-verified by several evaluators and had a small sample size. Notably, there may be differences between naturally occurring arthritis and experimentally induced model arthritis.

## Conclusions

This study was conducted to establish image evaluation grading criteria that CT imaging evaluation as a complement to X-ray evaluation for experimental stifle joint osteoarthritis model dogs. In conclusion, these image evaluation grading criteria may be used to evaluate the effects of exercise or drug treatments on arthritis lesions using a scoring system.

## Methods

The present study included 8 normal beagle dogs (Control group) and 24 surgical OA model beagle dogs four weeks post OA induction (OA group). Beagle dogs (12–18 months, male) were obtained from Xi’an Dilepu Biology and Medicine Co., Ltd. (Xi’an, China). All animal experiments were conducted in compliance with Animal Experimental Ethics Regulations noted and approved by the Institutional Animal Care and Use Committee (IACUC; Approved number 22-KE-616) in HLB bioStep Co., Ltd. (Incheon, Korea). Additionally, this study complied with the Animal Research: Reporting of In Vivo Experiments (ARRIVE) guidelines.

### Canine OA model

The beagle dog surgical OA model represents a fully functional load-bearing in vivo anatomical model for joint degeneration studies. The study included 8 male beagle dogs aged 12–18 months weighing approximately 10–12 kg. The dogs were thus skeletally mature and had body weights suitable for a weight-bearing animal model [18]. Prior to the surgical procedure, general anesthesia was intravenously induced using 0.1 ml/kg intravenous injection of Zoletil 50 (VIRBAC, France) and xylazine (Rompun®, Bayer AG, Germany,) in a 1:1 mixture, with additional injections given as needed. Isoflurane inhalation anesthesia was then administered via tracheal intubation. The region of both stifle joints was then prepped and painted with antiseptic povidone iodine and 70% alcohol. The ACTL procedure was then performed as previously described [19]. Briefly, ACLT was performed on the left hind limb, under aseptic conditions, with strict hemostasis but without using a tourniquet. A midline skin incision (3–4 cm) was made over the patellar ligament, and the skin and subcutaneous tissues were retracted medially and laterally to expose the medial parapatellar region. The capsule was incised, the joint was entered and examined to ensure that it was normal, and the anterior cruciate ligament was directly visualized. Any blood vessels seen on the surface of the ligament were cauterized. A sickle-bladed knife was passed posterior to the ligament, and the ligament was transected. The cut ends of the ligament were cauterized, and the capsule and skin were then closed. The procedure for the sham ACLT was the same, except that the ligament was not manipulated, cauterized, or cut.

### Manual joint palpation

All dogs underwent palpation of the treated limbs as described in previous studies on the OA model four weeks after the induction of OA (Table 3). One veterinarian evaluated pain on palpation of the stifle joint of each dog using a five-point scale (0–4) [20].

**Table 3:** Manual palpation grading criteria

Grade	Description
0	Does not notice palpation
1	Orients to site on palpation, does not resist palpation
2	Orients to site, may lick, slight objection to palpation (body tensing or splinting)
3	Moderate objection to palpation. Withdraws from palpation, may vocalize, may lick at site or pay attention to site after palpation. May become somewhat aggressive or show guarding behavior
4	Tries to escape palpation or prevent palpation, may bite, vigorously guards the area. May chew, bite, or rub area after palpation

### Gait analysis

Gait evaluation was performed according to the evaluation criteria described below on the OA model four weeks after the induction of OA (Table 4). One veterinarian evaluated pain on palpation of the stifle joint of each dog using a five-point scale (0–4) [21–23].

**Table 4:** Gait analysis

Grade	Description
0	Walks and trots normally
1	Slight lameness at walk or trot
2	Moderate lameness at walk or trot
3	Severe lameness at walk or trot
4	Extreme lameness (nonweight bearing) at walk or trot

### X-ray and computed tomography imaging

X-ray and computed tomography (CT) were taken in all dogs on the OA model four weeks after the induction of OA. General anesthesia was introduced using an intravenous injection of 0.1 ml/kg Zoletil 50 and xylazine in a 1:1 ratio mixture, with additional injections as needed. The anesthetized animals were placed on the table in the dorsal recumbency so that both stifle joints were positioned horizontally, and the defect region was examined by using CT (Scanner Alexion Model TSX-032A, Toshiba, Japan) and X-ray (MyVet Table X-500, Woorien, Korea). The severity of OA for all dogs was assessed using both X-ray and CT (Table 5). These grading criteria use a point scale ranging from 0 to 4, where 0 represents normal and 4 indicates the highest severity. After assessment, all the animals were moved to a warm and dry place for recovery. Vital signs were checked periodically during the experimental procedure and the recovery period.

**Table 5:** X-ray and CT imaging OA severity

OA severity	Description
0	No radiographic features of osteoarthritis
1	Doubtful opacity in infrapatellar fat pad and possible osteophytic lipping
2	Definite osteophytes and mild opacity in infrapatellar fat pad
3	Multiple osteophytes, moderate opacity in infrapatellar fat pad
4	Large osteophytes, severe opacity in infrapatellar fat pad

### Statistical analysis

The analyses were performed with IBM SPSS (version 29.0, IBM Corp, Armonk, New York, USA). The Mann‒Whitney U test was used to compare the results of manual joint palpation, gait analysis, and OA severity of the control group and OA group. Subsequently, Spearman’s rank correlation test was used to investigate the association between manual joint palpation, gait analysis, and OA severity. The significance level was set at *P* < 0.05.

## Data Availability

Data are available upon reasonable request to the corresponding author.

## Abbreviations

ACLT: Anterior cruciate ligament transection
ARRIVE: Animal research: reporting of in vivo experiments
CT: Computed tomography
IACUC: Institutional animal care and use committee
KL: Kellgren–Lawrence
OA: Osteoarthritis
